# Endocrine Late Effects in Childhood Cancer Survivors

**DOI:** 10.3390/cancers14112630

**Published:** 2022-05-26

**Authors:** Paula Casano-Sancho, Ana Carolina Izurieta-Pacheco

**Affiliations:** 1 CIBER de Diabetes y Enfermedades Metabólicas Asociadas (CIBERDEM), Pediatric Endocrinology Unit, Institut de Recerca SJS 39-57, Hospital Sant Joan de Déu, University of Barcelona, Esplugues, 08950 Barcelona, Spain; 2 Oncology Department, Pediatric Cancer Center Barcelona, Hospital Sant Joan de Déu, University of Barcelona, 08950 Barcelona, Spain; anacarolina.izurieta@sjd.es

**Keywords:** childhood pediatric cancer, childhood cancer survivors, endocrine late effects, endocrine sequelae

## Abstract

**Simple Summary:**

Recent advances in cancer treatment have led to improved survival, with an exponential increase in sequelae among survivors. Around 50% of survivors will experience at least one hormonal disorder, with radiotherapy, hematopoietic stem cell transplantation, and alkylating chemotherapy being the most frequently related. Therefore, lifelong monitoring of childhood cancer survivors at risk is paramount. With this review, we describe in detail the most prevalent endocrine sequelae, considering new approaches such as proton beam therapy and immune-related endocrinopathies with the advent of precision oncology treatment. We hope to encourage oncologists and endocrinologists to develop early detection guidelines that minimize sequelae and have a positive impact on their quality of life.

**Abstract:**

Childhood cancer management has improved considerably over the years, leading to a significant improvement in survival of up to 80%. However, childhood cancer survivors are at the highest risk of developing sequelae resulting from treatment, with endocrine complications being frequently observed among survivors. Multiple predisposing factors for endocrine sequelae have been identified, including age at diagnosis, treatment received, radiation, tumor type, and genetic polymorphisms, which could explain the individual predisposition to develop drug toxicity. Novel agents targeting tumor growth and immune checkpoint inhibitors have recently become the cornerstone for the treatment of different cancers, triggering a myriad of immune-related endocrinopathies. Endocrine sequelae of cancer therapy will have an impact on not only childhood but also on the survival and quality of life of these highly complex patients. Therefore, lifelong monitoring of childhood cancer survivors at risk of endocrine diseases is paramount. Encouraging oncologists and endocrinologists to develop new follow-up and early detection guidelines that minimize sequelae among these patients has become a priority, promoting integration between pediatric and adult units since many sequelae may manifest only after years to decades of follow-up.

## 1. Introduction

In recent years, childhood cancer management has improved considerably, shedding light on pediatric survival and overall event-free rates. The last decade has seen significant advances not only in conventional treatment but also in the development of targeted therapy with the emergence of novel therapies such as immunotherapy and molecular therapeutics applied to pediatric cancer. However, the increase in life expectancy has revealed a high incidence of chronic diseases among cancer survivors [1]. Endocrine sequelae account for a high proportion of these sequelae, occurring even decades after cancer treatment, with 40–60% experiencing at least one endocrine disorder over the course of their lives [2]. Children seem to be especially susceptible to developing these sequelae, with an individual risk of long-term sequelae that critically depends not only on the oncological treatment received but also on multiple predisposing factors such as age at diagnosis, tumor type, and genetic polymorphisms, which could explain the individual predisposition to develop drug toxicity [2,3]. Current international guidelines state that lifelong monitoring of childhood cancer survivors (CCS) at risk of endocrine diseases is paramount [4].

In this review, we summarize the current knowledge on endocrine sequelae among CCS and delineate facts about the lifelong monitoring of these patients, encouraging oncologists and endocrinologists to develop new follow-up and early detection guidelines that minimize sequelae among these patients.

## 2. Childhood Cancer Survivor Cohorts

Scientific research on CCS is vast and shows the experience of different healthcare centers with long-term follow-up after treatment schemes are fulfilled. Mainly retrospective publications that collected the experience of a single center have been described so far, and in recent years, publications of large multicenter cohorts of survivors have been published. The different cohorts published have followed different methodologies to collect sequelae among CCS. Despite these methodological differences, cohorts of CCS report a comparable risk of medical problems, which strengthens the reliability of the observations reported [5].

The wide number of patients included ranges from 35,996 for the Childhood Cancer Survivor Study (CCSS) [6] to 43,909 for the Adult Life after Childhood Cancer in Scandinavia (ALISCCS) [7], 17,981 for the British CCSS or 4400 for the French–British Lymphoma and Solid Tumor Cohort/the French Childhood Cancer Survivor Study for Leukemia (LEA), and 1075 for the Swiss CCSS. The rate of lack of monitoring over time is as high as 29.4% for the British CCSS, 23.3% for the CCSS, and <1% for the ALICCS cohorts [6]. It is interesting to note that in order to improve reliable estimations and avoid bias, the CCSS compares the incidence of medical problems with a cohort of the survivors’ siblings, while the ALICCS compares their results with a comparable healthy population. On the other hand, the CCSS also collects data regarding lifestyle and sequelae treatment, adding information on the effects of long-term treatment. In addition, the Children’s Oncology Group (COG) performed case–control studies to determine the role of genetics and the environment associated with long-term sequelae among CCS.

The CCSS cohort evaluated young adults between 17 and 34 years and found that at least 69% of patients followed-up had one relevant medical condition, 36% had two or more medical sequelae, and 27% had a severe medical condition that involved disability [8]. As a result of the evaluation of thousands of patients, in 2003, the COG published the “Long-Term Follow-up Guidelines for Survivors of Childhood, Adolescent, and Young Adults Cancers (COG-LTFU Guidelines)” [9], making specific recommendations based on scientific evidence about long-term follow-up in childhood cancer survivors, being reviewed periodically by multidisciplinary working groups.

## 3. Endocrine Sequelae among Childhood Cancer Survivors

Endocrine disorders are one of the most frequent sequelae described in CCS. The highest risk is seen in survivors of central nervous system (CNS) tumors, leukemias, bone tumors, and Hodgkin’s disease. In 2013, a report from the St. Jude Lifetime Cohort Study determined that 62% of 1713 CCS presented adverse endocrine-reproductive late effects [9]. The prevalence of chronic health conditions was 95% at 45 years, and at least 80% had a disabling chronic condition, with endocrine sequelae being one of the most prevalent conditions after cardiopulmonary diseases [6]. Moreover, endocrine disorders have implications as well in terms of long-term cardiometabolic morbidity, affecting at least 18% of all survivors [6].

We describe below the most common endocrine sequelae. To facilitate their description, we analyzed them according to the different treatments received (Table 1 and Table 2).

## 4. Radiotherapy

Endocrine late effects are most often observed in CCS that were previously exposed to radiation that included the hypothalamic–pituitary (HP) axis, thyroid, or gonads, and are mainly related to the total dose received, as well as the duration of treatment. Important considerations may include the method of delivery of radiation, as well as the radiation field [10].

### 4.1. Cranial Radiotherapy

Cranial radiotherapy (CRT) is the predominant risk factor for HP late effects, with anterior pituitary hormones such as GH, LH/FSH, TSH, and ACTH being potentially affected, leading to a significant increase in the risk of secondary sequelae [2]. In contrast to injury from tumor growth or surgery, where patients frequently present with HP disorders from the onset, radiation-induced HP dysfunction may appear even several decades after CRT [11]. Chemaitilly et al. [12], in a cohort of CCS who had HP radiation exposure, observed that at least 50% had one anterior pituitary hormone deficiency and 11% had multiple pituitary hormone deficiencies. The most common childhood CNS tumors that require radiotherapy include medulloblastoma, ependymoma, craniopharyngioma, and low- and high-grade gliomas, though other common childhood malignancies such as acute lymphoblastic leukemia with CNS involvement may also require CRT [13].

#### 4.1.1. Proton Beam Therapy

Proton beam (PB) therapy is the latest advancement in radiation therapy, as an alternative to conventional photon (X-ray)-based methods of irradiation, and has the potential to decrease further exposure of normal tissues located in proximity to the treated tumor [14]. So far, it is limited to only a few healthcare centers, with only a small number of studies and almost scarce long-term follow-up.

With the advent of PB radiotherapy, there has been considerable optimism about its potential to reduce endocrine late effects in childhood brain tumor survivors. In 2011, Viswanathan et al. [15] carried out the first study comparing endocrine outcomes between patients treated with PB radiation only and a group receiving combined conventional plus PB radiation therapy. They observed a high rate of radiation-induced HP dysfunction in children with brain tumors who received either PB radiotherapy alone (47%) or combined conventional and PB irradiation (33%), suggesting that children who undergo treatment with PB radiation therapy are also at high risk of developing HP hormone deficiencies [13,16]. In 2016, Eaton et al. [17], in a multi-institutional cohort study that included children with standard-risk medulloblastoma treated with chemotherapy and proton or photon radiation between 2000 and 2009 with endocrine screening follow-up, demonstrated a statistically significant association between the use of PB and reduced incidence of hypothyroidism and gonadal deficiency, a reduced need for endocrine replacement therapy, and greater height at last follow-up among matched cohorts of medulloblastoma survivors. As the authors highlighted, one limitation of the study is that they did not define whether the hypothyroidism or sex hormone deficiency was primary or central (HP axis deficiency). As the craniospinal irradiation doses are similar in both treatments, we can hypothesize that the dose delivered directly to the thyroid and gonads, as well as to the HP axis, is reduced with PB therapy, as previously described [18].

Recently, Aldrich et al. retrospectively studied a large cohort of patients with medulloblastoma and defined the lower risk of primary hypothyroidism after proton therapy, but the overall risk of HP deficiency (ACTH/GH deficiency) was similar between radiation modalities [19].

There is still scarce information to make a conclusion on whether PB therapy reduces endocrine long-term sequelae. Further studies are still needed.

#### 4.1.2. Growth Hormone (GH) Deficiency

GH deficiency (GHD) is the most common hormonal disorder and the earliest that appears after CRT in brain tumor survivors. Its severity and speed onset are dose dependent and increase with time elapsed since irradiation [14]. GHD has well-documented negative effects related not only to poor growth and abnormal body composition but also to poor overall health, increased cardiovascular risk, and cognitive impairment that relies on diminished quality of life [20,21]. Radiotherapy regimens that use at least 8–12 Gy yield are associated with a 25% incidence of GHD at 10 years of follow-up; meanwhile, this incidence increases up to 80–100% when we use doses higher than 30 Gy [22,23]. Prospective studies also suggest that abnormal GH responses to provocative tests can occur as early as 3 months and certainly in the first 12 months after CRT, and the incidence tends to increase with time of follow-up, stating that increasing cranial radiation dose and time after treatment are the main risk factors for developing GHD [24,25]. Doses higher than 30 Gy may result in HP dysfunction, yet lower doses may affect GHD secretion [2]. Additionally, clinical studies of GH secretion after CRT have shown that the HP axis of the young is more radiosensitive than that of adults, with age at time of radiotherapy being an additional clinical predictor of GHD. Therefore, reducing hypothalamic irradiation should be a priority when treating children with brain tumors if the target is not adjacent to the hypothalamus or when proton therapy can be used to reduce the impact of irradiation [23].

The protocolized follow-up of CNS tumor survivors has allowed optimizing treatment of GHD with recombinant human growth hormone (rhGH). Although there have been continued concerns that GH therapy may increase the risk of tumor recurrence and secondary neoplasms, especially meningiomas and gliomas, further analysis has correlated this risk with the fact of having received CRT [26]. Even though there are only a few studies in this regard, the current recommendation is to start rhGH after at least one year of complete cancer treatment to avoid early neoplasm recurrence [4,27]. Treatment with rhGH is effective in increasing height, improving about 10–20 cm in final height [24,28].

#### 4.1.3. Gonadotropin Deficiency

Gonadotropin deficiency prevalence is reported to be 6.5% in overall cancer survivors and up to 11% if there is a history of HP axis radiotherapy [10]. It manifests in a range of severities, from subtle and subclinical abnormalities with sex hormones at the lower limit of normality to severe deficiencies in circulating sex hormone levels. Children and adolescents may experience pubertal delay. In adulthood, it can be manifested by arrested puberty, primary or secondary amenorrhea, or symptoms related to low estrogen or testosterone levels. Endocrinologists should be aware of these long-term complications in order to recognize them early, especially in men when subtle forms of gonadotropin deficiencies are present [29]. HP tumors and CRT doses ≥30 Gy within or near the HP region are the main risk factors for gonadotropin deficiency [10,29], but an association with lower irradiation doses of 20 Gy may be seen with extended follow-up [10]. Diagnosis follows the same guidelines as in non-cancer patients.

#### 4.1.4. Early Puberty

Low doses of cranial irradiation employed in the central nervous system prophylaxis in ALL may cause early puberty, which predominantly affects girls. When these doses increase up to 30 Gy, there is a similar incidence between boys and girls [30]. The GnRH pulse generator is highly radiosensitive, as low-dose irradiation causes precocious puberty and might be caused by damage to inhibitory GABAergic mechanisms, leading to disinhibition and premature activation of GnRH neurons [31], whereas high-dose irradiation is associated with delayed sexual maturation.

#### 4.1.5. Hyperprolactinemia

Hyperprolactinemia is frequently observed in CCS, as it represents direct radiation-induced damage to pituitary lactotroph cells, frequently observed with high doses of irradiation ≥30–40 Gy, and presentation may vary from years to decades after CRT [2]. There is reduced hypothalamic release of the inhibitory neurotransmitter dopamine [28]. Prolactin levels are often mildly elevated and may normalize within time. It is highly recommended to measure prolactin levels on clinical suspicion [1].

#### 4.1.6. Adrenocorticotropin (ACTH) Deficiency

ACTH deficiency (ACTHD) is the most life-threatening HP deficiency, with nonspecific symptoms in an unstressed state that may include anorexia, nausea, shakiness relieved by eating, hypoglycemia, poor weight gain, or easy fatigability and hypotension, as well as even death from adrenal crisis [32], and may be the result acutely following surgery where the hypothalamus or the pituitary gland is affected [2]. Following irradiation, up to 30% of patients develop ACTHD and may be diagnosed even decades after CRT, with increasing risk when other functional defects of the HP axis are present [33]. Highlighting the correlation between irradiation dose and ACTHD, Crowne et al. [34] reported no damage to the HP–adrenal axis following lower doses of 18–24 Gy in the treatment of ALL after 3.6 to 10 years of follow-up compared with higher doses of radiation used in the treatment of brain tumors when ACTHD was identified after a similar period of follow-up [33,35]. Hence, ACTHD is associated with radiation doses ≥30 Gy [1]. Diagnosis becomes challenging in such patients because the insulin tolerance test (ITT) is considered the gold standard; however, it is contraindicated in patients with a history of seizures, which immediately excludes a proportion of brain tumor survivors. The glucagon stimulation test has gained relevance by allowing the assessment of both GH and ACTH reserves, appearing to be comparable with the ITT test. Awareness of ACTHD in cancer survivors, especially brain tumor survivors, is essential, and monitoring should be continued beyond at least 10 years following CRT [36].

#### 4.1.7. Central Diabetes Insipidus

Diabetes insipidus is mainly an acute complication of tumor location affecting the hypophyseal area, with patients with histiocytosis, germinoma, craniopharyngioma, or as a result of neurosurgical procedure being at greater risk. Diabetes insipidus has not been consistently reported as a direct consequence of RT for sellar or extrasellar brain tumors. Symptoms of polydipsia, polyuria, and nocturia are the most frequent. Patients with hypothalamic involvement are at risk of a thirst regulation disorder. Inadequately low urine osmolarity despite high serum osmolarity (and/or hypernatremia) is diagnostic. Sometimes, to establish the diagnosis, a water deprivation test is needed. Treatment consists of desmopressin acetate administered intranasally or orally [37].

#### 4.1.8. Thyroid Hormone Deficiency

Primary hypothyroidism is more common in cancer survivors, although the cumulative incidence of central hypothyroidism (hypothalamic or pituitary) is high during the ten years after CRT and is considered a consequence of higher doses of irradiation (≥30 Gy) [38]. Recent studies show that TSH secretory dysregulation after CRT might precede other endocrine disorders in some patients previously not well recognized because of the use of only relatively insensitive tests such as single measures of TSH and T4 [34]. If TSH is not measured until GH deficiency becomes apparent, the diagnosis of hypothyroidism might be delayed. In children, missed growth opportunities and potential functional implications of undiagnosed hypothyroidism indicate the need for early intervention [39].

### 4.2. Neck and Thoracic Radiotherapy

The most common use of neck and thoracic radiotherapy in pediatric oncology is to treat Hodgkin’s disease (HD), with a non-negligible secondary risk of thyroid affection [4]. With doses of 10 Gy, the development of hypothyroidism is imminent, with a cumulative incidence of 28% for HD survivors, at least 4–5 times higher than that expected in the general population. The main risk factors for underactive thyroid remain dose of radiation, female sex, and older age at diagnosis [40]. Over time, hyperthyroidism, as well as a higher risk of thyroid neoplasms, has often been observed [5]. The incidence of thyroid nodules among HD survivors has varied from 2 to 65% [41], being identified even 20 years after radiotherapy [3]. Ensuring periodic testing of thyroid function, along with careful palpation of the thyroid gland, must be performed routinely and throughout the lifetime of cancer survivors [39].

### 4.3. Abdominal, Pelvic, and Genitourinary Radiotherapy

The testicular germinal epithelium is especially sensitive to radiation. Spermatogenesis can be impaired by direct testicular irradiation, including total-body irradiation (TBI), or because of other adjacent treatment fields such as pelvic or flank irradiation [42]. It is observed after testicular doses as low as 0.1 Gy with a secondary increase in FSH and seminogram alterations, and recovery is unlikely when exceeding 4–6 Gy [4,42]. Leydig cell insufficiency has been described with 20 Gy. More than 83% of cancer survivors treated with combined protocols that included chemotherapy and abdominal/pelvic radiotherapy or TBI may develop azoospermia [4]. However, 17% of them might recover spermatogenesis within 15 years after treatment [4], so long-term follow-up is strongly recommended [43]. It is paramount that healthcare providers are aware of the risk of impaired spermatogenesis, testosterone deficiency, and physical sexual dysfunction. Monitoring growth and pubertal development and progression is recommended for prepubertal and peripubertal survivors treated with radiotherapy exposing the testes to ≥12 GY or TBI, as well as evaluating gonadotropins and testosterone to diagnose hypogonadotropic hypogonadism and program hormonal treatment for pubertal induction. In postpuberal boys, it is strongly recommended to perform semen analysis, in addition to clinical evaluation of testicular volume and hormonal data such as follicle-stimulating hormone (FSH) and inhibin B, for identification of impaired spermatogenesis [43].

On the other hand, the potential exposure of the ovaries to pelvic radiotherapy, as well as combined treatment schemes with alkylating agents and other gonadotoxic treatment modalities, may lead to an increased risk of premature ovarian insufficiency (POI) [44]. POI can occur early, during, or immediately following cancer treatment completion, although frequently, it develops above years after completion of cancer treatment but prior to 40 years [42], and the prevalence is estimated to be at least 11% among CCS data reported in the largest cohort of female cancer survivors [45]. Abdominal radiation is also thought to damage the tail of the pancreas, leading to pancreatic endocrine insufficiency with an increased risk of glucose intolerance and diabetes mellitus in survivors [9,46].

## 5. Chemotherapy

### Gonadal Complications

Gonadal dysfunction remains one of the main long-term effects of chemotherapy, affecting not only endocrine but also reproductive function in CCS [4].

In males, exposure to gonadotoxic chemotherapy only, especially under high-dose therapy regimens entailing alkylating agents such as cyclophosphamide, procarbazine, chlorambucil, or platinums, may lead to mild subclinical testosterone deficiency, but Leyding cell function is generally safeguarded [2]. However, exposure to combined regimens that involve not only moderate doses of chemotherapy but also radiation may lower the threshold for Leydig cell damage [2,47]. Germ cell toxicity may lead to oligospermia and azoospermia, in correlation with the cumulative doses. Cumulative doses exceeding ≥7.5 g/m^2^ cyclophosphamide or ≥60 mg/m^2^ ifosfamide are associated with permanent azoospermia [4,46]. Follow-up of these patients is highly recommended, as recovery from azoospermia may be possible after years [2]. Inhibin B appears to be a promising biochemical marker, having a higher correlation with spermatogenesis than FSH4, and prospective studies in childhood cancer survivors are required to determine the correlation with future fertility [48].

Meanwhile, in women, ovarian tissue appears to be less sensitive than testicular tissue, although equally, this risk is increased if combined chemotherapy and radiotherapy regimens are used. Cytotoxic agents inhibit follicular growth, affecting the ovarian follicular reserve [45]. Hence, ovarian involvement might affect puberty, increasing the risk of PFO or impaired fertility [4], with risk of amenorrhea that increases from 0 to 83% in reported series, and the most important risk factors being the cumulative dose of alkylating agents and older age at treatment. Furthermore, there is also an increased risk of pregnancy-related complications such as abortion, low birth weight, and premature birth [2]. Anti-Müllerian hormone (AMH) and FSH levels can be used to assess the antral follicle count to estimate ovarian reserve, although FSH values may vary in cancer survivors; AMH seems to be well correlated with estimation of ovarian reserve [48]. Nonetheless, the correlation between AMH and fertility has not been established yet, and future studies are needed.

## 6. Hematopoietic Stem Cell Transplantation (HSCT)

Endocrinological sequelae are the most frequent after HSCT due to multifactorial reasons. Rescue therapies that involve cumulative effects of previous chemotherapy received, as well as the use of high-dose steroid therapy and TBI, are the main causes of developing endocrinological sequelae, with an actual risk of up to two and three times higher than other oncologic patients. They present hypogonadism (83%), hypothyroidism (56%), and GH deficiency (50%), with a frequency even higher than patients who received CRT. HSCT is the main risk factor predisposing to growth alterations, as well as overweight and metabolic risk.

## 7. Multifactorial Endocrine Sequelae

### 7.1. Calcium and Phosphate Metabolism

Childhood cancer survivors are at higher risk of developing decreased bone mineral density (BMD), mainly those who received treatment with methotrexate, corticosteroids, or HSCT [1]. The best method for screening BMD in pediatric age is still under debate. According to COG guidelines, it is recommended to perform a DEXA scan in high-risk survivors when entering the follow-up program and repeat if clinically indicated. The worldwide accepted Z-score to evaluate BMD in adults is not validated in pediatric age, so there is no consensus on the values for recommendation of treatment in these cases. To reduce long-term fractures, it is highly recommended to encourage patients to engage in physical activity, as well as optimize calcium intake and normal vitamin D levels in CCS [49,50].

### 7.2. Obesity

There are not enough series to collect data that directly establish a cause–effect between obesity and oncological treatments. The reported prevalence varies between 18 and 25%, even lower than that reported in the youth population of the geographical area. Patients at higher risk are those who received CRT ≥ 20 Gy, women, or children ≤ 4 years who received radiation therapy. Moreover, 26% develop insulin resistance and almost 23% dyslipidemia with increased metabolic risk [51,52].

### 7.3. Metabolic Syndrome and Diabetes

Metabolic syndrome and diabetes increase long-term mortality in CCS. The risk of diabetes is described in almost all cohorts of cancer survivors. The most prevalent mechanism is probably related to insulin resistance and obesity, categorized as diabetes type 2. Few groups have studied the prevalence of autoantibodies in these patients that can lead to autoimmune endocrinopathy. The prevalence of autoantibodies in these patients seems to be exceptional [53]. Some therapies such as abdominal radiation induce direct pancreatic insufficiency [2]. TBI seems to induce insulin resistance rather than pancreatic damage and may induce other endocrinopathies that contribute to the development of diabetes. Risk factors associated with the development of metabolic syndrome are CRT, TBI, use of corticosteroids, and obesity. The prospective follow-up of these patients over the years must include health education involving participation in physical activities and risk factor modulation [53,54].

## 8. Future Research

### 8.1. Novel Targeted Agents

Advances in cancer therapy over the last few years have included the development and actual wide use of medications that modulate immune checkpoint proteins [55]. Endocrinopathies are one of the most common side effects of these emerging therapies, including hypophysitis, thyroid dysfunction, and the development of diabetes mellitus [50]. ACTH and/or TSH deficiency are the most common pituitary hormone abnormalities described in patients with hypophysitis related to immunotherapy [56]. Adrenal insufficiency is usually permanent, needing lifelong steroid replacement. Secondary hypothyroidism recovery may vary within series (6–64%) [51,57].

Tyrosine kinase inhibitors (TKIs) have become more widespread in use as targeted therapies in a variety of tumors. Endocrine-related side effects associated with these agents include alterations in thyroid function (hypothyroidism, hyperthyroidism, and thyroiditis), bone metabolism in response to dysregulation of calcium and phosphate metabolism developing hypocalcemia, and 25-OH vitamin D deficiency with secondary parathormone (PTH) elevation, linear growth, gonadal function, fetal development, glucose metabolism, and adrenal function [58,59].

### 8.2. Chimeric Antigen Receptor (CAR) T-Cell Therapy

CAR-T therapy is a novel therapy for relapsed or refractory leukemias. There are limited data on endocrine side effects. Because this therapy remains to be reserved for patients who have already received multiple schemes of conventional chemotherapy or HSCT, we should consider these patients at the highest risk of developing secondary endocrine side effects [60].

### 8.3. Preservation of Fertility in Prepubertal Boys

In postpubertal boys’ sperm, cryopreservation before starting gonadotoxic treatment is a usual procedure to preserve fertility due to the high sensibility of Leydig cells to the damage of chemotherapy and radiation. In prepubertal boys, sperm cryopreservation is not an option; therefore, in patients with high risk of gonadal toxicity, new experimental approaches are under investigation [61]. Testicular tissue freezing is a new option for these patients, but we should note the experimental nature of the interventions [59]. Experimental research is focused on three approaches: spermatogonial stem cell propagation and autotransplantation, testicular tissue autografting, and in vitro spermatogenesis. In the future, endocrinologists should pursue these new approaches and experimental methods, with the possibility that they will become part of clinical practice involving prepubertal boys [59,62].

### 8.4. Preservation of Fertility in Female Cancer Patients

Female patients who are at potential risk of infertility due to ovarian RT, HSCT, and use of high-dose alkylating agents should be offered an informed choice about fertility preservation. For postpubertal girls, oocyte or embryo cryopreservation is an established method. Young adults may likely opt for oocyte cryopreservation, as they are less likely to be partnered with or interested in sperm donors. Hormone stimulation is needed for oocyte retrieval within 2 weeks of stimulation. If treatment cannot be delayed for 2 weeks, ovarian tissue cryopreservation is an alternative [63,64].

Ovarian tissue cryopreservation is the only method for fertility preservation that is available for prepubertal girls and postpubertal women who cannot undergo oocyte stimulation. The technical risks of ovarian tissue resection are small and related to the laparoscopic technique and anesthesia and may be performed at the same time as other surgeries (i.e., central venous catheter placement). It is important that patients and their families understand the limitations that are associated with the future use of ovarian tissue [64,65].

In patients with leukemia and other malignancies with risk of ovarian metastasis, the use of ovarian cryopreservation is limited, and there is a real concern for the oncologist about the risk of reimplantation of affected tissue. This is an area of ongoing research. The use of techniques of in vitro maturation of excised ovarian tissue may be an option in the near future for these patients [66].

## 9. Conclusions

The data published so far of cohorts of CCS manifest the urgent need to establish a mandatory prospective follow-up of these patients. In the last decade, with the efforts made so far, we have seen an improvement in the diagnosis and treatment of different sequelae after oncological treatment. The active participation of different medical specialists optimizes the long-term care of these patients, improving their quality of life and survival rate.

The establishment of multidisciplinary units of CCS is mandatory for the optimal care of these patients. Although this approach has improved over time, currently, not all hospitals treating CCS have created specific follow-up units. The endocrine sequelae described in this review may appear in the long term after completion of oncological treatments. Therefore, an important objective in the near future is to design transition units to continue adult care for these patients. In addition to continuing hormonal replacement, prospective screening programs for new endocrinopathies are needed. The objective will be to involve adult endocrinologists in their long-term follow-up and promote collaboration between childhood and adult units.

Metabolic and hormonal control is crucial for the long-term survival of CCS, as increased cardiometabolic risk has been described in these patients. Patient education, prevention, and lifestyle measures are important to improve survival, with continuity throughout the lifetime.

## Figures and Tables

**Table 1:** Endocrine sequelae after childhood cancer therapies.

Treatment Received	Hypothalamic–Pituitary Axis				Gonads	Thyroid	BMD *	Metabolic Syndrome
	GH	LH/FSH	TSH	ACTH				
Cranial Radiotherapy (RT) α	GH deficiency (>10 Gy)	Precocious puberty (10–30 Gy) Delayed puberty (>30 Gy) Hypogonadism (>30 Gy)	Central hypothyroidism (>30 Gy)	Central adrenal insufficiency (>30 Gy)	-	-	-	-
Abdominal RT ∞	-	-	-	-	Gonadal failure	-	-	Metabolic syndrome/diabetes
Neck RT ≠	-	-	-	-		Nodules/hypo/hyperthyroidism/thyroid cancer	-	-
Alkylating Agents	-	-	-	-	Gonadal failure/Leydig cell dysfunction	-	-	-
Heavy Metals	-	-	-	-	Gonadal failure/Leydig cell dysfunction	-	-	Dyslipidemia
Antimetabolite	-	-	-	-	-	-	Low BMD *	-
Total-Body Irradiation	√	√	√	√	√	√	Low BMD *	Obesity/metabolic syndrome
Immunotherapy	Hypophysitis	√	√	√ Central adrenal insufficiency	Hypogonadism	Hypothyroidism	-	Diabetes
Tyrosine Kinase Inhibitors	-	-	-	-	-	Hypothyroidism/thyroiditis	Hypocalcemia/vitamin D	-

α Cranial irradiation includes: cranial, orbital/eye, ear/infratemporal, and nasopharyngeal. ∞ Abdominal RT: Gonadal radiation includes: lumbosacral spine, abdomen, and pelvis (females) and pelvis and testicular (males). ≠ Neck RT: thyroid irradiation includes: thyroid, neck, cervical spine, oropharyngeal, supraclavicular, mantle, and mini-mantle. * Bone mineral density.

**Table 2:** Endocrine sequelae in the most frequent childhood cancers.

Type of Tumor	Gonadal	HP Axis	Thyroid ^1^	Adrenal	BMD	Obesity
ALL/AML/Relapse	QT alkylating agents QT nitrosoureas Testicular RT	Craniospinal RT (selected cases) HSCT TBI	TBI	Glucocorticoids	Glucocorticoids MTX HSCT TBI	Glucocorticoids TBI HSCT
NH and Hodgkin’s Lymphoma	QT alkylating agents Abdominal RT	-	Cervical RT	Glucocorticoids	Glucocorticoids MTX	Glucocorticoids
CNS Tumors	QT alkylating agents	Surgery Craniospinal RT	-	-	-	Surgery Craniospinal RT
Osteosarcoma	QT alkylating agents QT Platin agents	-	-	-	MTX	-
Ewing Sarcoma	QT alkylating agents QT platin agents RT (depends on localization)	-	-	-	-	-
Neuroblastoma	QT alkylating agents Abdominal RT	-	131I-MIBG ^131^I-labeled monoclonal antibody	Surgery	-	-

^1^ Thyroid: hypothyroidism, thyroid nodules, differentiated thyroid neoplasms. HP: hypothalamic–pituitary axis; BMD: bone mineral density; CNS: central nervous system; ALL: acute lymphoblastic leukemia; AML: acute myeloid leukemia; NH: non-Hodgkin’s lymphoma; QT: chemotherapy; RT: radiotherapy; HSCT: hematopoietic stem cell transplantation; TBI: total-body irradiation; MTX: methotrexate; 131I-MIBG: iodine-131-metaiodobenzylguanidine.

## Abbreviations

CCS	Childhood cancer survivors
CNS	Central nervous system
HP	Hypothalamic–pituitary axis
CRT	Cranial radiotherapy
PB	Proton beam therapy
TBI	Total-body irradiation
GHD	Growth hormone deficiency
rhGH	Recombinant human growth hormone
IGF-1	Insulin-like growth factor 1
TSH	Free-thyroxine (fT4) thyroid-stimulating hormone
LH	Luteinizing hormone
FSH	Follicle-stimulating hormone
AMH	Anti-Müllerian hormone
ACTHD	ACTH deficiency
ITT	Insulin tolerance test
BMI	Body mass index
HD	Hodgkin’s disease
LLA	Acute lymphoblastic leukemia
POI	Premature ovarian insufficiency
HSCT	Hematopoietic stem cell transplantation
BMD	Bone mineral density
TKI	Tyrosine kinase inhibitors
CAR-T	Chimeric antigen receptor T-cell therapy
